# A multicentre registry of hospitalized patients with acute and chronic heart failure: Study design of the H^2^‐registry

**DOI:** 10.1002/ehf2.15266

**Published:** 2025-04-13

**Authors:** Johannes Leiner, Sebastian König, Anne Nitsche, Sven Hohenstein, Jana Nagel, Melchior Seyfarth, Henning Baberg, Alexander Lauten, Hans Neuser, Alexander Staudt, Jürgen Tebbenjohanns, René Andrié, Michael Niehaus, Markus W. Ferrari, Ralf Kuhlen, Andreas Bollmann

**Affiliations:** ^1^ Department of Electrophysiology Heart Centre Leipzig at University of Leipzig Leipzig Germany; ^2^ Real World Evidence and Health Technology Assessment at the Helios Health Institute Berlin Germany; ^3^ Study Centre Leipzig at the Helios Health Institute Leipzig Germany; ^4^ Department of Cardiology University Hospital Wuppertal, University of Witten/Herdecke Wuppertal Germany; ^5^ Department of Cardiology and Nephrology Helios Hospital Berlin‐Buch Berlin Germany; ^6^ Department of Cardiology Helios Hospital Erfurt Erfurt Germany; ^7^ Department of Cardiology and Angiology Helios Hospital Plauen Plauen Germany; ^8^ Department of Cardiology and Angiology Helios Hospital Schwerin Schwerin Germany; ^9^ Department of Cardiology and Intensive Care Helios Hospital Hildesheim Hildesheim Germany; ^10^ Department of Cardiology, Rhythmology and Electrophysiology Helios Hospital Siegburg Siegburg Germany; ^11^ Department of Cardiology Helios Hospital Gifhorn Gifhorn Germany; ^12^ Clinic for Internal Medicine I: Cardiology and Intensive Care Helios Dr. Horst Schmidt Hospital Wiesbaden Wiesbaden Germany; ^13^ Helios Health Institute GmbH Berlin Germany; ^14^ Fresenius SE & Co. KGaA Bad Homburg v.d. Höhe Germany

**Keywords:** Chronic heart failure, Acute heart failure, Patient registry, Patient‐reported outcomes, Hospitalization, Mortality, Guideline‐directed medical therapy

## Abstract

**Aims:**

Heart failure (HF) is a highly prevalent condition affecting 1–3% of the adult population in Europe. Despite landmark improvements in HF care over the last two decades, hospitalization and mortality rates remain relatively high. Gathering real‐world data on HF populations is crucial, especially in the light of newly emerging therapeutic approaches. The Helios Heart (H^2^)‐registry was established to provide up‐to‐date, real‐world data on a contemporary cohort of hospitalized HF patients in Germany using a standardized set of outcome measures with a focus on patient‐reported outcomes (PROs). This manuscript describes the registry's design and presents an interim analysis of baseline characteristics and 1‐year outcomes.

**Methods and results:**

The H^2^‐registry is a prospective, investigator‐initiated, multicentre observational registry in Germany that started in 2021 and is actively enrolling patients. Inpatients ≥18 years of age with a present diagnosis of chronic or acute HF are recruited in secondary and tertiary hospitals throughout Germany. Routine follow‐up (FU) is conducted every 6 months. Data collection is based on a set of variables following recommendations of the International Consortium of Health Outcome Measurements (ICHOM) covering data on demographics, medical history, HF characteristics, medication, procedures, and patients' perceived health status via the collection of standardized PROs. Until 31 December 2023, a total of 2361 patients were enrolled in 10 study centres. Mean age in this cohort is 72 years, 36.9% are female, and median left ventricular ejection fraction is 45%. An analysis of 6‐month and 12‐month outcomes in a cohort of 1593 patients with complete FU data revealed all‐cause mortality rates of 9.0% and 16.2% at 6 and 12 months, while HF‐related rehospitalizations occurred in 24.4% and 43.5% at 6 and 12 months.

**Conclusions:**

The H^2^‐registry is currently the largest ongoing prospective registry of HF patients in Germany. It is foreseeable that the H^2^‐registry will significantly contribute to the collection of real‐world data and provide a comprehensive and unique perspective on the current characteristics, treatment strategies, and resulting outcomes of HF patients in Germany.

Trial registration number: NCT04844944.

## Graphical abstract

The H^2^‐Registry: overview, first results and outlook.

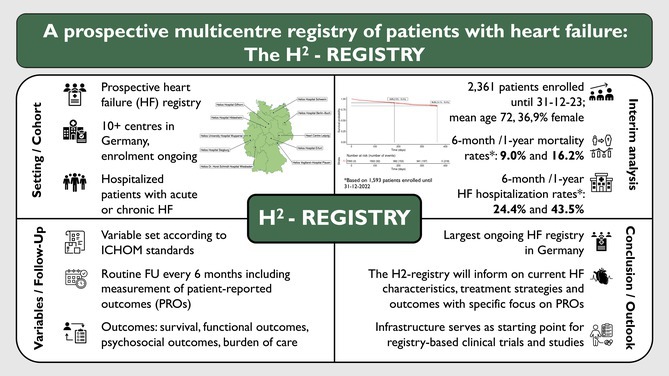

## Introduction

Heart failure (HF) is a major public health issue and has frequently been termed as global pandemic with an estimated prevalence of 1–3% in developed countries^1^, ^2^, ^3^ and increasing disease burden.^4^, ^5^ Patients suffer from a pronounced decline in quality of life and are exposed to a high mortality and hospitalization risk.^5^, ^6^, ^7^

Advances regarding pharmacological treatment approaches that were shown to be highly effective in randomized controlled trials (RCTs) led to a paradigm shift in HF therapeutic management in the last decade with corresponding guideline recommendations for medical therapy.^1^, ^8^ While for patients with chronic heart failure with reduced ejection fraction (HFrEF), there are four well‐established substance classes with a class I guideline recommendation^1^ (i.e. renin–angiotensin–aldosterone system inhibitors [RAASi], beta‐blockers, mineralocorticoid receptor antagonists [MRA], and sodium‐glucose cotransporter‐2 inhibitors [SGLT2i]), pharmacological treatment for chronic heart failure with mildly reduced ejection (HFmrEF) and preserved ejection fraction (HFpEF) is more challenging for clinicians with limited options. In a recent guideline update issued by the European Society of Cardiology (ESC), SGLT2i received a class I recommendation for treatment of HFmrEF and HFpEF,^9^ opening up novel treatment options also in these patient cohorts.

In order to improve generalizability beyond selected HF patient cohorts and to gain further insights from the study of large datasets, real‐world evidence (RWE) and in this context multicentre real‐world registries are of particular importance to complement evidence derived from RCTs and to investigate the implementation of recent guideline recommendations.^10^, ^11^, ^12^, ^13^ Numerous HF registries have been established in recent years. From a European perspective, large continent‐wide HF registries led by the ESC such as the ESC Heart Failure Long Term Registry (ESC‐HF‐LT)^6^ and the ESC‐HF III registry^13^ should be mentioned as prime examples. Additionally, the Swedish Heart Failure Registry^11^ represents an example for a nationwide population‐based quality registry. From a more global perspective, the Global Congestive Heart Failure Registry (G‐CHF) aims to provide and compare data on HF patients from different geographical regions including developing countries.^14^ REPORT‐HF is another international registry focusing on patients with an index hospitalization for HF.^15^

Over the past years, these mentioned studies generated crucial RWE and improved our understanding of HF patient characteristics and clinical phenotypes, its clinical trajectory, and unveiled findings regarding the real‐world implementation of interventional and medical therapies, which ultimately advanced the quality of HF patient management.^6^, ^7^, ^16^, ^17^, ^18^, ^19^, ^20^, ^21^, ^22^, ^23^, ^24^, ^25^

Despite the highly significant burden of disease with an estimated national prevalence of ~4%,^5^, ^26^ HF patients in Germany are inconsistently represented in international HF registries. Both the ESC‐HF‐LT and ESC‐HF III registries did not include German centres^6^, ^13^; however, there is a German contribution for G‐CHF and REPORT‐HF.^14^ Currently, no ongoing HF registry exists in Germany. Evidence derived from national registries is very limited with only a small number of published studies, which furthermore entail limitations regarding representability, aim, and data topicality.^27^, ^28^, ^29^ This highlights the urgent need to generate prospective and up‐to‐date real‐world data on HF cohorts in Germany in order to further characterize patients and assess clinical outcomes and treatment implementation.^30^, ^31^

Significant heterogeneity exists between HF registries in view of data collection and choice of study variables.^32^, ^33^ Especially, standardized measurement of functional and psychosocial status and in general disease‐associated quality of life (QoL) via patient‐reported outcomes (PROs) is carried out inconsistently in the above‐mentioned registries. The International Consortium for Health Outcome Measurement (ICHOM),^34^ a not‐for‐profit organization, has defined and published a set of HF outcome measures based on expert consensus and taking into account a survey of variables collected in previously conducted large HF registries.^35^ Based on this publicly available variable set including standardized patient‐reported outcome measures (PROM) tools, we designed the multicentre German Helios Heart (H^2^)‐registry to generate critical and urgently needed RWE and ultimately contribute to HF patient management. To the best of our knowledge, the H^2^‐registry is the first study to apply the ICHOM standards for HF. In this manuscript, we describe the design of the H^2^‐registry and present an interim analysis of baseline characteristics and one‐year outcomes of patients enrolled until 31 December 2023.

## Methods

### Aim, objectives, and study endpoints

Based on ICHOM recommendations,^35^ the H^2^‐registry was designed with the main objectives:

- to describe clinical profiles, outcomes, and therapeutic strategies in hospitalized HF patients,
- to assess current patient management and consistency with the ESC HF guidelines 2021 (with its focused update issued in 2023)^1^, ^9^ focusing guideline‐directed medical therapy (GDMT), and
- to collect comprehensive PRO data on HF patients over the course of their disease and to identify associations between therapeutic interventions and PROs.

A detailed overview of surveyed endpoints is provided in *Table* *1*. There is a particular focus on patients with HFpEF and HFmrEF to investigate the impact of therapeutic interventions on long‐term outcomes as explanatory endpoints, as there is a paucity of real‐world data in this context. Of note, the H^2^‐registry was initially set up as a general registry for cardiovascular diseases (CVD) including atrial fibrillation and coronary artery disease. Inclusion of patient with these conditions using the established registry infrastructure will soon follow. At present, the H^2^‐registry only considers HF patients.

**Table 1:** Endpoints of the H^2^‐registry

All‐cause mortality during FU
Cardiovascular mortality during FU
In‐hospital mortality (index hospitalization)
Length of hospital stay (index hospitalization)
Rate of rehospitalizations for HF during FU
Rate of rehospitalizations for other specific cardiovascular diseases during FU (myocardial infarction/ischaemic heart disease, stroke or transient ischaemic attack, peripheral arterial disease, venous thromboembolism or pulmonary embolism, atrial fibrillation, other cardiovascular causes)
General quality of life (PROMIS GH‐10, PHQ‐2), baseline and FU
Disease‐specific quality of life (KCCQ‐12), baseline and FU
Rate of prevalent and incident comorbidities, baseline and FU
Rate of prescribed drug treatment stratified according to class of drug, baseline and FU
Adherence to medical therapy during FU
Adverse events associated with medical therapy, baseline and FU
Rate of performed cardiovascular interventions stratified according to procedure type, baseline and FU
Adverse events associated with HF device therapy, baseline and FU

FU, follow‐up; HF device therapy, cardiac resynchronization therapy with or without defibrillator, implantable cardioverter defibrillator therapy; HF, heart failure; KCCQ‐12, Kansas City Cardiomyopathy Questionnaire; PHQ‐2, Patient Health Questionnaire‐2; PROMIS GH‐10, Patient Reported Outcomes Measurement Information System Global Health‐10.

### Design

**Figure 1:**
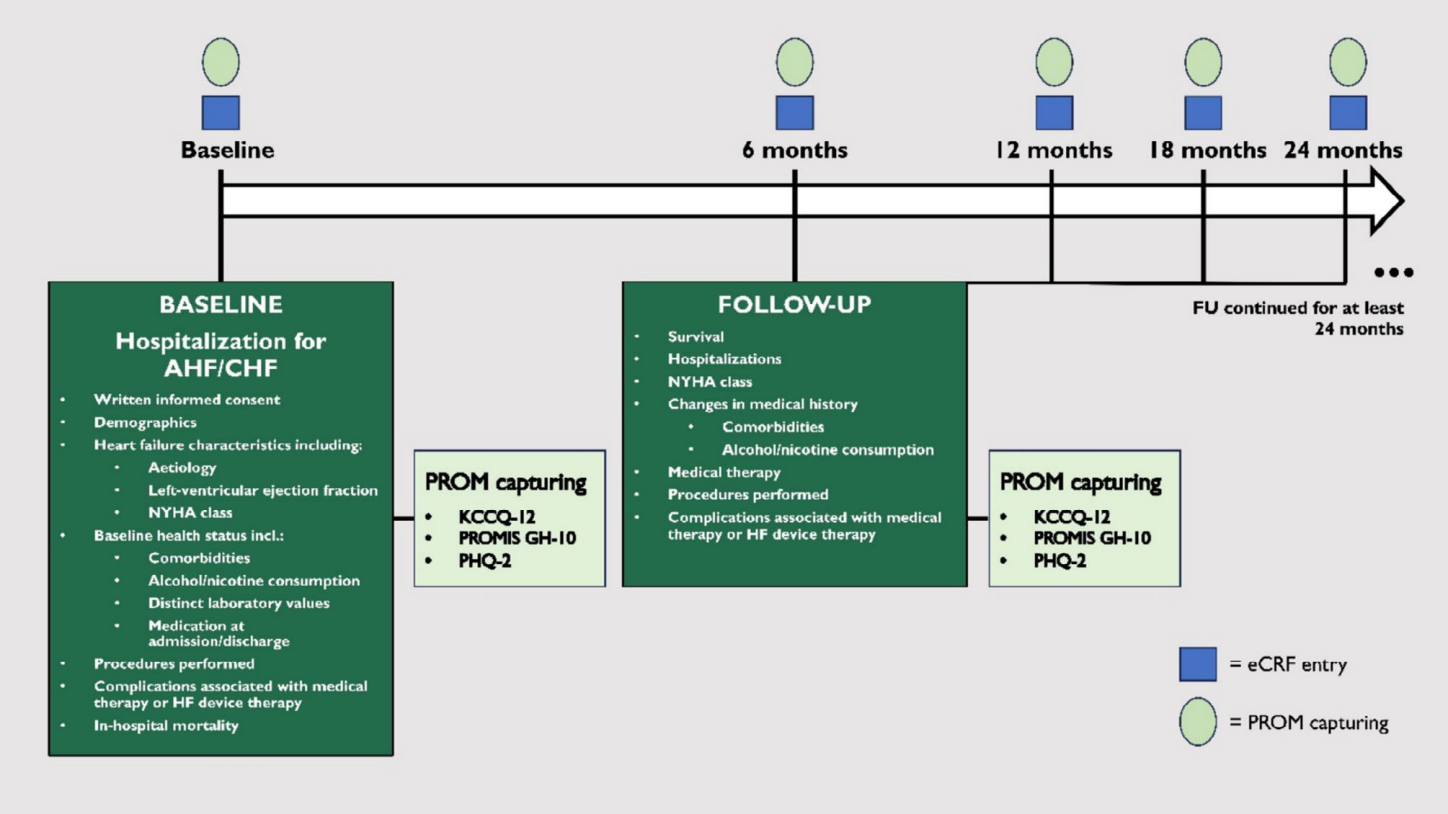
Overview of data collection time points and variables. AHF, acute heart failure; CHF, chronic heart failure; eCRF, electronic case report form; FU, follow‐up; HF, heart failure; KCCQ‐12, Kansas City Cardiomyopathy Questionnaire; NYHA, New York Heart Association; PHQ‐2, Patient Health Questionnaire‐2; PROM, patient‐reported outcome measure; PROMIS GH‐10, Patient Reported Outcomes Measurement Information System Global Health‐10.

The H^2^‐registry ( *clinicaltrials.gov* identifier: NCT04844944) is a prospective, investigator‐initiated, multicentre observational registry focusing on inpatients with guideline‐adherent HF diagnosis. Enrolment takes place during an inpatient stay (index hospitalization) in the cardiology units of the participating study sites in Germany. The reason for the patient's index hospitalization is not considered an inclusion criterion but is documented, it can be an elective or emergency admission for HF or for other causes. Routine follow‐up (FU) is performed every 6 months with no formal restrictions on FU duration. *Figure* *1* summarizes the time points of data collection with corresponding variables. Ethical approval for this study was given by the ethics committee of the University of Leipzig (AZ 116/21‐ek) and by local ethics committees at each participating site. The H^2^‐registry is conducted according to the requirements of ‘good clinical practice’ as well as to the principles outlined in the Declaration of Helsinki 1975.

### Setting

**Figure 2:**
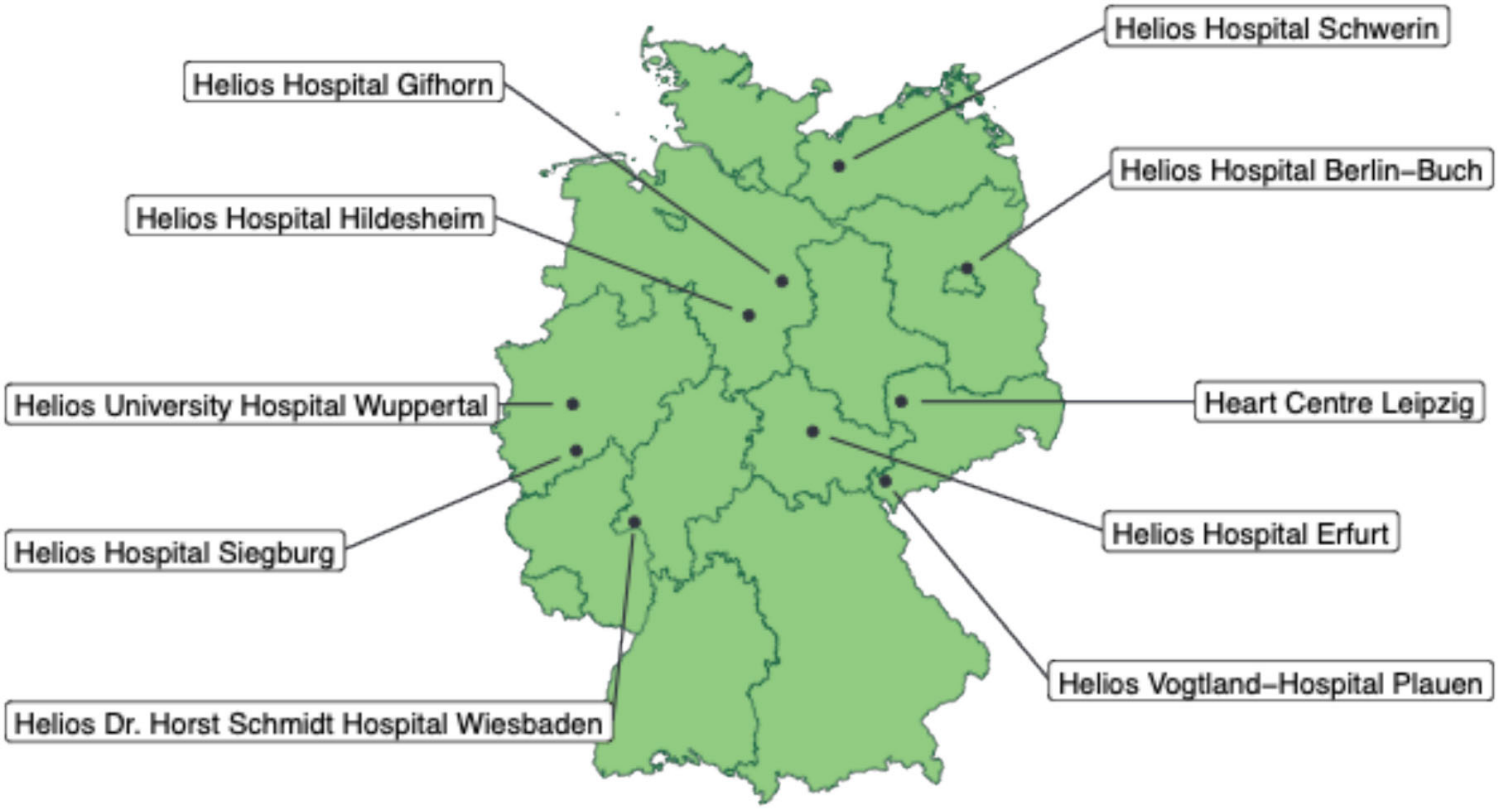
Study centres.

Ten active study centres (secondary and tertiary hospitals) of the Helios hospital network across Germany are currently enrolling patients for the H^2^‐registry (*Figure* *2*). The Helios network is Germany's largest healthcare provider, consisting of acute care hospitals and outpatient clinics. The scientific leadership and oversight of the H^2^‐registry lie with the Helios Health Institute (HHI), with its Leipzig site adjacent to the lead study centre, the Heart Centre Leipzig University Hospital. Study centres were appointed by the principal investigator and co‐investigators at HHI according to their size (large cardiology unit required) and available resources for trial conduction (i.e. availability of site‐specific investigators and study nurses). The data analyses are conducted by biostatisticians at the HHI.

### Population and consent

Enrolment started on 30 March 2021 and recruitment is ongoing. Recruitment and FU are expected to be completed by 2027, with the aim of enrolling at least 4000 patients with at least 24 months FU duration each. The last study centre was initiated in June 2022. By 31 December 2023, 2361 patients with HF had been enrolled (for specifics, please see ‘Results from interim analysis’ section). A detailed written informed consent, signed by the patient and the site‐specific investigators, is provided to each patient during index hospitalization. Informed consent is documented electronically within the software *i.s.h.med®* (*SAP SE, Walldorf, Germany*), and every patient is assigned a master patient index number.

### Inclusion and exclusion criteria and patient selection

Inclusion criteria are as follows:

- Age of 18 years or older
- Inpatient treatment
- Present diagnosis of chronic or acute HF regardless of ejection fraction according to the definitions by current ESC guidelines^1^, ^9^

Exclusion criteria are as follows:

- Inability to sign written informed consent
- Initial presentation (index hospitalization) in cardiogenic shock or other kinds of shock
- Patients who previously underwent heart transplantation or patients with present ventricular assist device

Eligibility of every patient is determined by site‐specific investigators (i.e. physicians with sufficient training in the field of cardiology). To avoid selection bias, investigators and study personnel are provided each day with a randomized sample of 5–10 patients (with a probable HF diagnosis based on the International Statistical Classification of Diseases and Related Health Problems [ICD‐10]‐encoded admission diagnosis or discharge diagnoses from previous hospital stays) admitted during the previous 24 h. The HF diagnosis (as defined in the current ESC HF guidelines^1^) of patients within this sample is then confirmed by the investigators, following a thorough review of clinical, instrumental findings (i.e. echocardiography or additional imaging studies such as stress echocardiography, laevocardiography or magnetic resonance imaging, 12‐lead electrocardiogram, chest x‐ray) and laboratory findings (N‐terminal pro‐B‐type natriuretic peptide [NT‐proBNP]). The diagnosis of HFpEF is additionally verified by applying the HFA‐PEFF diagnostic algorithm, where a score of ≥5 points clearly suggests HFpEF.^36^ Enrolment takes place during the first 2 days of the index hospitalization.

### Baseline and follow‐up data

Selection of baseline and FU variables is based on recommendations by ICHOM with its specifics published previously.^35^ In brief, a pragmatic variable set was developed through first surveying the specifics of existing HF registries followed by a collaborative decision‐making process within an expert working group. A standardized set of baseline variables, treatment variables, and outcome measures covering four main domains (survival, functional outcomes, psychosocial outcomes, burden of care) is proposed by the working group. For a holistic outcome measurement in HF, the ICHOM working group emphasizes the necessity of using validated PROM tools. Three PROMs are included in the recommendations, consisting of the HF‐specific Kansas City Cardiomyopathy Questionnaire (KCCQ‐12),^37^ PROMIS GH‐10 (Patient Reported Outcomes Measurement Information System Global Health‐10)^38^ with two summary scores (physical health and mental health) assessing the patients' general health perception, and the Patient Health Questionnaire‐2 (PHQ‐2),^39^ an ultra‐short screening instrument for depression. ICHOM provides a publicly available data dictionary that includes relevant variables as well as recommendations for the time points of data collection.^35^, ^40^, ^41^ The variable set was slightly adjusted according to investigator consensus to serve the requirements for the H^2^‐registry. For example, the coverage of HF‐related procedures and medical therapy has been extended to adequately reflect the recommendations of the ESC guidelines. A detailed overview of collected variables can be found in the Appendix (*Tables* *A1* *and* *A2*). For the collection of baseline data of the index hospitalization, all patient information available in the hospital information system at admission, during in‐hospital course, and at discharge is considered relevant.

### Data capture and storage

Baseline and FU data are entered into an electronic case report form (eCRF) (*secuTrial, interActive Systems GmbH, Berlin, Germany*) by investigators or designated trained study personnel at each study centre. Most data are entered manually; however, numeric data like height, weight, or laboratory values are extracted from the hospital information systems automatically. If information from available medical records is insufficient, direct patient interviews are conducted by study personnel during the index hospitalization. For PROMs, paper‐based questionnaires are handed out to each patient during the index hospitalization. An optical character recognition software (*ABBYY, Charlotte, NC, USA*) is used to facilitate PROM data capturing. PROM scores are calculated automatically through implemented algorithms within the study database according to the specifications of each questionnaire.^37^, ^38^, ^39^

Routine FU every 6 months including PROM capturing is planned and carried out by each participating site. Patients usually receive the three PROM questionnaires and an additional questionnaire assessing the patients' medical history during the past 6 months including changes in oral medication, hospitalizations, and performed procedures (for collected variables, see *Table* *A2*) via mail. Patients are contacted via telephone if no response is registered after 2 weeks or if the provided information is not sufficient. Furthermore, as the H^2^‐registry aims to establish a digitalized infrastructure,^42^ patients are offered an option for digital FU entries via the web patient interface of *secuTrial*. A solid data privacy concept in compliance with federal data protection regulations including the double‐pseudonymization of patients' data for central access at the HHI is guaranteed.

### Data quality

**Figure A1:**
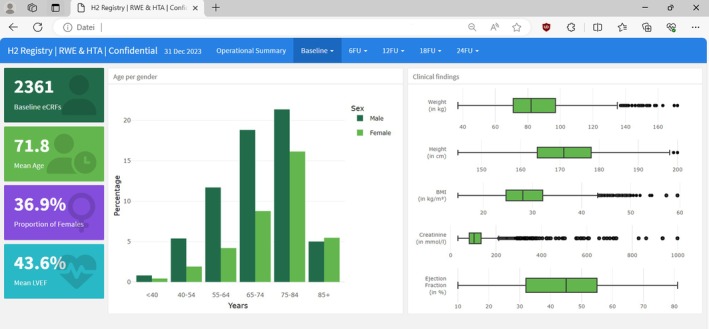
Monthly dashboard.

Quality assurance monitoring of eCRF data is established for the H^2^‐registry. A designated monitor of the HHI is regularly performing remote as well as on‐site monitoring according to a pre‐defined monitoring plan. Data entries are reviewed for completeness, plausibility, and conformity with the study protocol. The eCRF software itself has a built‐in alert system with notifications in the case of incomplete data to enhance completeness of data entries. These reviews are supported by regular plausibility checks of eCRF data performed by HHI data scientists who produce monthly interactive dashboards displaying the collected data, which correspond to interim analyses (see *Figure* *A1*).

### Statistical methods

For the interim analysis presented in this manuscript, baseline characteristics were displayed in a descriptive manner. For survival analysis, we applied the Kaplan–Meier method. PROM scores were calculated using questionnaire‐specific methods.^37^, ^38^, ^39^ Of note, for calculation of T‐scores for PROMIS GH‐10, we applied an established method derived from a US reference population.^38^, ^43^ For statistical comparison of PROM scores, we applied a Student's *t*‐test.

## Results from interim analysis

### Baseline characteristics

**Figure A2:**
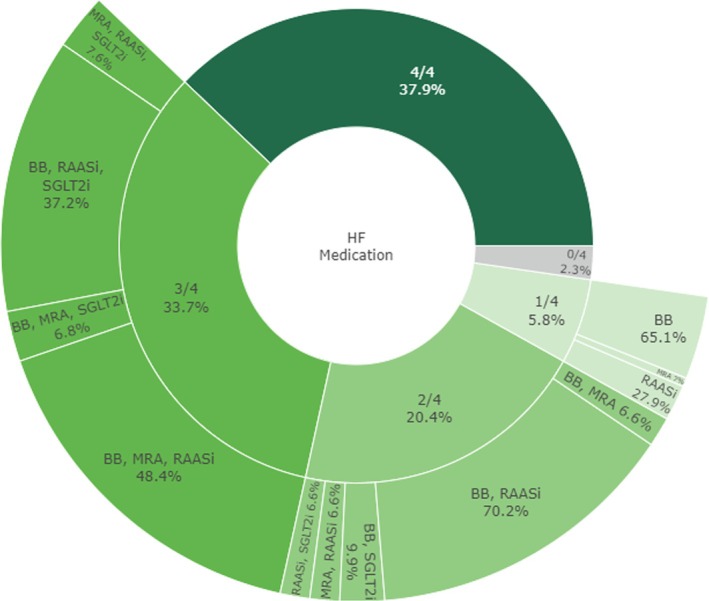
HF medication among HFrEF patients at hospital discharge.

By 31 December 2023, 2361 patients had been enrolled in 10 study centres. Median age in this cohort is 74 years, 36.9% are female, and median left ventricular ejection fraction (LVEF) is 45%. According to the current guideline definition,^1^ HFrEF is present in 37.9%, HFmrEF in 20.7%, and HFpEF in 41.4%. Co‐existing ischaemic heart disease was present in 39.7% of patients at baseline, and most patients were in New York Heart Association (NYHA) stage III. The index in‐hospital mortality is 1.3%, including patients with chronic and acute HF. In *Table* *2*, baseline characteristics of HF patients included in the H^2^‐registry are shown. *Table* *A3* summarizes the patients' oral medication including changes of prescription during index hospitalization. RAASi and beta‐blockers were the most commonly prescribed substance classes at hospital discharge. SGLT2i were most frequently initiated during the inpatient course (13.2%). Corresponding to the high prevalence of atrial fibrillation, 57.1% received oral anticoagulants (mainly non‐vitamin K anticoagulants). Among HFrEF patients, 37.9% received all four substance classes with a class I ESC guideline recommendation^1^ at hospital discharge (i.e. present therapy with RAASi, MRA, beta‐blockers, and SGLT2i; *Figure* *A2*).

**Table 2:** Baseline characteristics of HF patients included in the H^2^‐registry up to 31 December 2023

Age (years), median [IQR]	74 [65–81]
Female, %	36.9
Left ventricular ejection fraction (LVEF) in %, median [IQR]	45 [32–55]
Body mass index (BMI) in kg/m^2^, median [IQR]	28 [24.6–32.1]
Creatinine in mmol/L, median [IQR]	103 [82–134]
HFrEF, %	37.9
HFmrEF, %	20.7
HFpEF, %	41.4
NYHA class II, %	31.2
NYHA class III, %	46.6
NYHA class IV, %	10.8
Previous HF hospitalization, %	66.8
Ischaemic heart disease, %	39.7
Hypertension, %	86.7
Atrial fibrillation, %	58.7
Renal dysfunction, %	52
Diabetes mellitus, %	38.1
Vascular disease, %	31.2
Emergency admission, %	52.8
Elective admission, %	39.8
Index case in‐hospital mortality, %	1.3

HF, heart failure; HFmrEF, heart failure with mid‐reduced ejection fraction; HFpEF, heart failure with preserved ejection fraction; HFrEF, heart failure with reduced ejection fraction; IQR, interquartile range; NYHA, New York Heart Association.

At baseline, mean ± standard deviation (SD) KCCQ‐12 summary score was 53.47 ± 24.63, reflecting a poor to fair HF‐related health status.^37^ Mean PROMIS GH‐10 physical health T‐score was 37.91 ± 6.78 and mean PROMIS GH‐10 mental health T‐score was 44.05 ± 7.03. Compared to a US reference population, this reflects a poor to fair physical health and a rather good mental health among H^2^‐registry patients.^44^ Mean PHQ‐2 score was 1.79 ± 1.75, suggesting a rather low degree of depressive symptomatology.^39^

**Figure A3:**
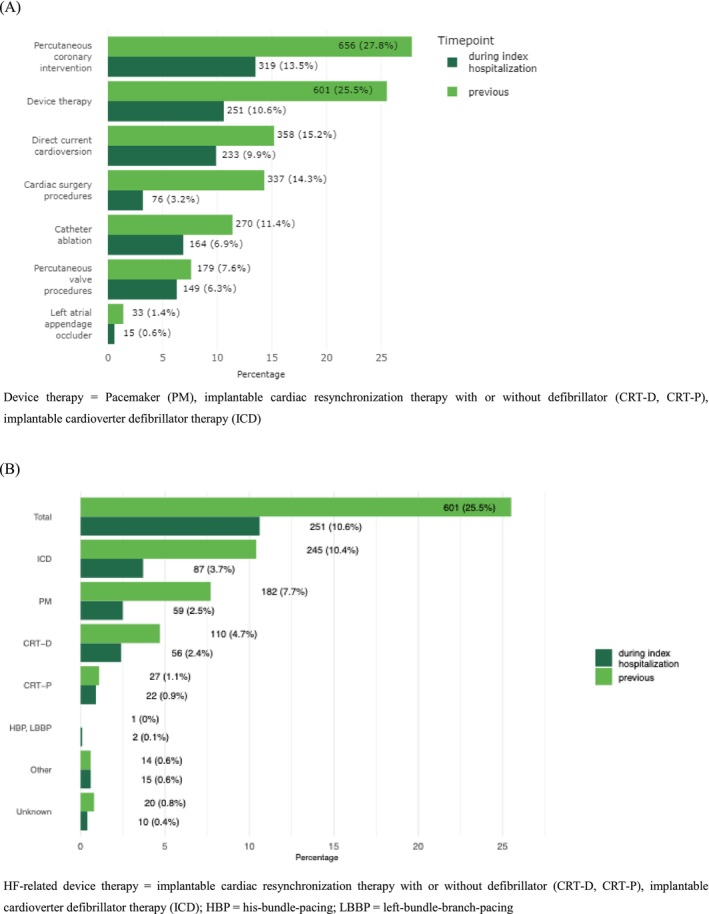
Cardiovascular interventions performed on H^2^‐registry patients during index hospitalization or in previous medical history.

With regard to cardiovascular interventions, percutaneous coronary intervention (PCI) was the most common procedure performed on HF patients in the H^2^‐registry during the index hospitalization or in their previous medical history, followed by device therapy (*Figure* *A3* *A*). An HF‐related cardiac implantable electronic device (i.e. cardiac resynchronization therapy with or without defibrillator, implantable cardioverter defibrillator therapy) was implanted in 165 (7%) patients during index hospitalization, and 382 (16.2%) of all patients had a previously implanted HF‐related device (*Figure* *A3* *B*).

### One‐year outcomes

To report on mortality and HF‐related rehospitalizations at 6 and 12 months, we performed an analysis including patients with at least 12 months of FU period. Only patients enrolled before 31 December 2022 and with available FU information of at least one FU visit were considered with no differentiation between acute and chronic HF. Patients were excluded if they were lost to FU before the first FU. Patients were censored at the day of their last FU or at 365 days.

**Figure 3:**
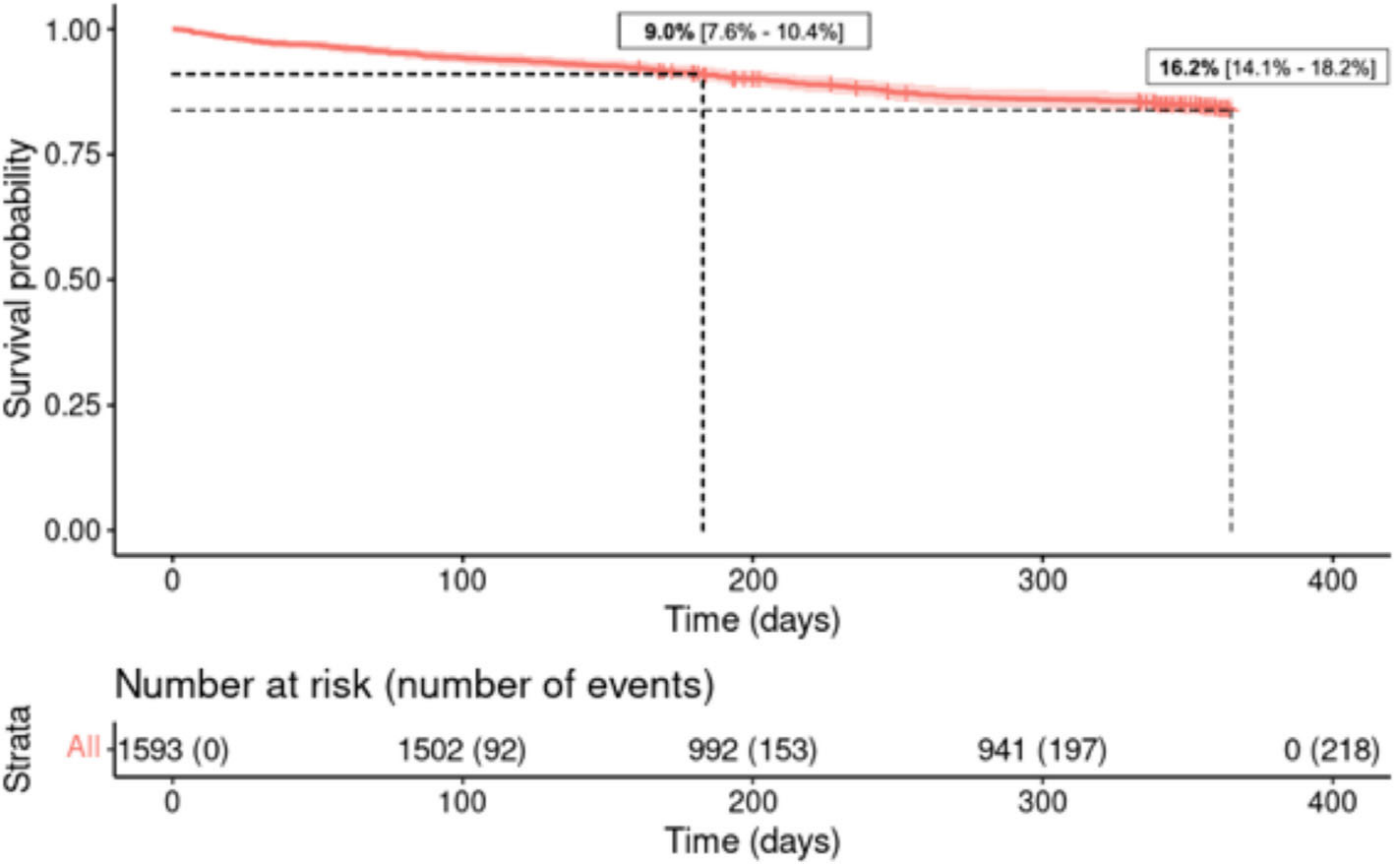
Kaplan–Meier survival curve for time to all‐cause mortality. Only patients included until 31 December and with available FU information of at least one FU visit were included. Patients who were lost to FU before the 6‐month FU were excluded. Patients are censored at the date of the last FU or at 365 days. The cohort includes 1593 patients. Mortality rates at 6 and 12 months are shown in the diagram.

The outcome cohort consisted of a total of 1593 patients (female: 36.1%; median age: 72.3). All‐cause mortality rates (with 95% confidence interval [CI]) at 6 and 12 months were 9.0% [7.6–10.4%] and 16.2% [14.1–18.2%] (*Figure* *3*). HF‐related rehospitalizations occurred in 24.4% at 6 months and 43.5% at 12 months, while hospitalizations due to other cardiovascular causes occurred in 8.6% and 15.0%, respectively.

**Figure A4:**
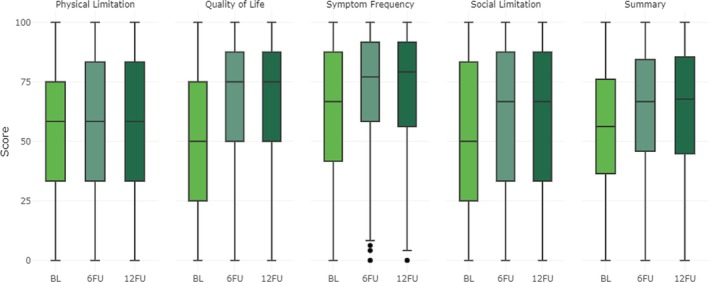
KCCQ‐12 scores at baseline, 6‐month, and 12‐month follow‐up.

**Figure A5:**
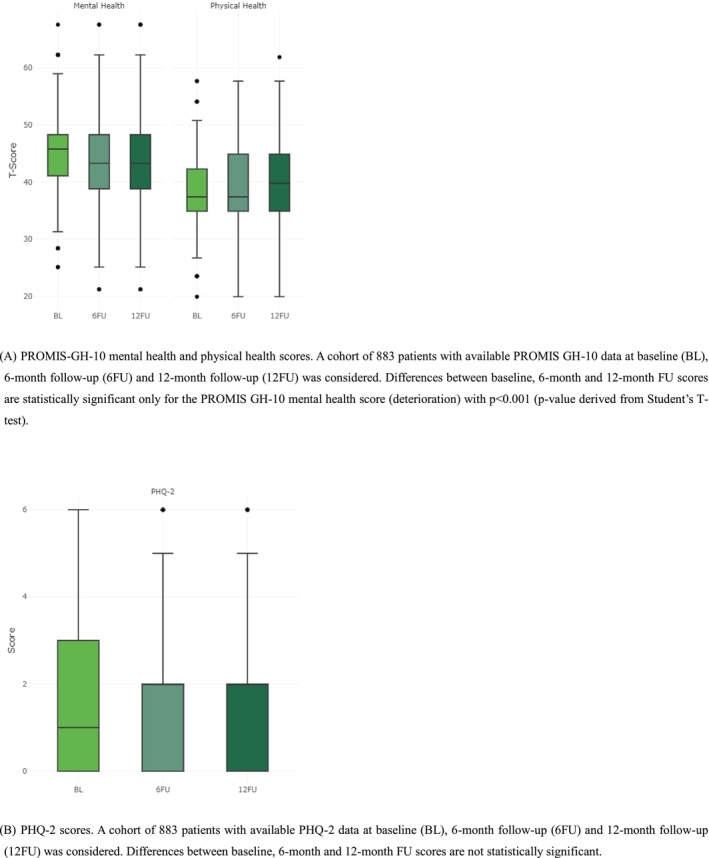
PROMIS GH‐10 (A) and PHQ‐2 (B) scores at baseline, 6‐month, and 12‐month follow‐up.

Patient‐reported, HF‐related health status improved following index hospitalization with regard to KCCQ‐12 scores. In a separately analysed cohort with available PROM data at baseline, 6‐month FU, and 12‐month FU consisting of 878 patients, mean KCCQ‐12 summary score was 55.77 ± 24.62 at baseline and improved to 63.68 ± 24.89 at 6 months and 63.68 ± 25.42 at 12 months (*P* value derived from a Student's *t*‐test baseline vs. 6‐month and baseline vs. 12‐month FU: <0.001). Furthermore, we observed a significant difference in all KCCQ‐12 subscales (*Figure* *A4*). A slight deterioration of mental health scores derived from PROMIS GH‐10 was observed, while there were no differences in PROMIS GH‐10 physical health scores and PHQ‐2 scores (*Figure* *A5*).

## Discussion

The management of CVDs represents one of the major challenges of healthcare systems worldwide, while HF is a highly prevalent clinical syndrome within the CVD spectrum associated with high risk for adverse outcomes and significant decline in QoL. Whereas clinicians now have the possibility to apply an improved range of therapeutic interventions, some important challenges remain for HF patient management. For example, medical and interventional treatment options in distinct HF subgroups (HFpEF, HFmrEF) are still limited. Additionally, implementation of GDMT remains inadequate, as findings from large‐scale HF registries have shown,^45^, ^46^, ^47^, ^48^, ^49^, ^50^, ^51^, ^52^ which ultimately affects patients' outcomes.^48^, ^53^

With the H^2^‐registry, we have established the infrastructure for a nationwide HF registry with a target enrolment of 4000 patients, while by the end of 2023, 2361 patients with acute and chronic HF have already been enrolled in 10 active study centres. Our registry will provide comprehensive data on demographics, treatment strategies, and outcomes of HF patients with focus on survival, burden of care, and PROs. Following standardized ICHOM recommendations for variable collection, we perform FU every 6 months and aim for a minimum FU duration of 24 months in each patient. With enrolment starting in 2021 and still ongoing, the H^2^‐registry will cover a period after publication of the recent ESC HF guidelines and its focused update^1^, ^9^ with the introduction of SGLT2i as elementary component of medical therapy in HF patients. The H^2^‐registry will provide critical data regarding the real‐world implementation of HF medical (and interventional) therapy and highlight subsequent influences on patients' outcomes. Baseline data showed that only 37.9% of HFrEF patients received complete GDMT at index hospital discharge (*Figure* *A2*). This rate is comparable to numbers reported from the Swedish Heart Failure Registry^50^ or REPORT‐HF.^49^ However, previously reported rates of optimal GDMT in HFrEF patients from North America are even lower.^46^, ^51^, ^52^ Of note, the mentioned trials mostly report on the use of RAASi, MRA, and beta‐blockers, as patient data before the publication of guideline recommendations for SGLT2i in the treatment of HFrEF were analysed and data on the real‐world use of SGLT2i are scarce. However, with rather low rates of optimal GDMT, the H^2^‐registry may come with certain limitations in reflecting the paradigm shift in HF therapy with regard to patient outcomes.

Among large international HF registries, standardized capturing of PROs varies significantly between the different studies. The ESC‐EORP‐LT and Swedish Heart Failure registries do not report on PROs,^6^, ^11^ while the ESC‐HF III registry^13^ collects PRO data with use of the non‐HF‐specific European Quality of Life 5 Dimensions (EQ‐5D) questionnaire.^54^ Among the above‐mentioned registries, only G‐CHF and REPORT‐HF assess HF‐related health status using the validated KCCQ‐12.^37^ Using baseline measurements from the KCCQ‐12 in over 23 000 patients, therefore creating one of the largest PRO databases in HF patients, the G‐CHF registry investigators were recently able to demonstrate significant variation in QoL across geographic regions and its strong independent association with mortality and hospitalization risk.^7^ In REPORT‐HF, serial measurements of the KCCQ‐12 at baseline and during FU unveiled evidence that a high proportion of patients after a hospitalization event for acute HF continue to experience poor QoL up to 12 months after the index event.^55^ This highlights the importance of evaluating therapeutic interventions not only in selected RCT cohorts but also in real‐world settings and moreover stimulates discussion on the inclusion of QoL as a routinely measured outcome parameter in HF patients.^55^ Although representing an important endpoint in clinical trials, PROMs are rarely used in clinical HF care; however, published data supporting this observation are sparse.^56^, ^57^ In current European guidelines, PRO assessment is not particularly mentioned as a component of HF patient management^1^ as opposed to other CVDs like atrial fibrillation, where routine PRO measurement is recommended by ESC guidelines with a class I indication.^58^ The American HF guidelines recognize the benefit of standardized PRO assessment to provide additional information on the patient's health status, which can ultimately guide therapy.^8^ However, data regarding benefits of PRO‐based HF patient management are limited.^8^, ^56^, ^57^

The H^2^‐registry will provide closely tracked PRO data on HF patients with data collection time points every 6 months allowing for a comprehensive characterization and assessment of patients' health status during over the course of the disease, particularly with regard to therapeutic interventions. Although the non‐interventional study design does not allow the effect of PRO‐guided management of HF patients to be investigated, observations from the H^2^‐registry might highlight the importance of a regular survey of patients' perceived health status and incorporating these findings into therapeutic decisions.

Our presented data revealed a relatively high proportion of HFpEF patients in comparison to previously described HF cohorts.^18^, ^22^, ^49^, ^59^, ^60^ However, there is a significant variation in HFpEF prevalence by geographical region, and trends towards increasing prevalence are evident.^21^, ^61^, ^62^ A better understanding and recognition of the disease and improved diagnostic criteria over the last years^1^, ^36^ may explain the distribution in HF phenotypes observed in the H^2^‐registry.

Our interim analysis of 1‐year outcomes in a cohort of patients with both acute and chronic HF showed high unadjusted mortality and rehospitalization rates at 1 year after the index hospitalization. Within the cohort of ESC‐HF‐LT registry all‐cause mortality rates are reported to be 23.6% for acute HF and 6.4% for chronic HF.^6^ Among 41 061 outpatients with chronic HF enrolled in the Swedish Heart Failure registry, 1‐year mortality rates ranged between 15.4%, 14.2%, and 17.4% for HFrEF, HFmrEF, and HFpEF patients, respectively,^60^ overall comparable to our observed 1‐year mortality estimate (16.2%). We noted a high HF‐related rehospitalization rate at 1 year of FU (43.5%), which is considerably higher in comparison to data from previously published registry‐based studies. Among outpatients enrolled in the ESC‐HF‐LT registry, the HF hospitalization rate at 1 year was 12.4%^59^ (mean age 64.8 years). In the REPORT‐HF registry, which enrolled patients with an index hospitalization for acute HF, a rehospitalization rate at 1 year of 22% among the overall cohort of 18 102 patients (median age 67 years) and 26% in a subcohort of patients treated in high‐income countries (median age 71 years) was noted.^63^ This trend in HF rehospitalization needs to be further monitored during the FU of the H^2^‐registry, and predictors of mortality and hospitalization will be identified in further analyses.

We noted an improvement in perceived health status and HF‐related quality of life as measured by KCCQ‐12. Baseline mean KCCQ‐12 summary score was lower in comparison to published data from the G‐CHF registry, where a mean KCCQ‐12 summary score of 62.5 was reported for patients in western European countries.^7^ Future analyses will focus on identifying associations between therapeutic interventions and QoL improvement and subsequent effects on mortality and hospitalization rates.

### Limitations

We recognize several limitations associated with the H^2^‐registry. As only inpatients are included during a hospitalization event, the H^2^‐registry cannot report on characteristics and outcomes of patients treated exclusively in the outpatient setting. Furthermore, a selection bias towards patients with advanced disease (who require hospitalization) is likely. Of note, we decided to include all HF patients who met the inclusion criteria, regardless of the reason that led to index hospitalization. As some patients are admitted electively for certain cardiovascular procedures, this may also result in a higher number of documented procedures compared to other HF cohorts.

Participating study centre hospitals are all part of the Helios hospital network, which might suggest a certain bias with regard to patient selection. However, the German health insurance system allows any patient to be treated in Helios hospitals regardless of the type of health insurance (statutory or private) or insurance company. The study centres represent hospitals with large cardiology departments, high levels of experience, and high standards of care. Therefore, we acknowledge as a limitation that the therapeutic standards and patient outcomes might differ from those in more peripheral hospitals. *Figure* *2* shows the overall good geographical distribution of the study centres, which are located in seven different federal states, although it must be acknowledged, that the registry does not adequately cover southern Germany. Furthermore, we acknowledge the rather low number of study centres in comparison to other multinational or national HF registries.^6^, ^11^, ^13^, ^14^, ^15^ Although the Heart Failure Association of the ESC recently formulated a data collection standard for HF for use in observational and interventional studies, there is still significant heterogeneity regarding variable collection between different HF registries.^32^, ^33^ We decided to base the selection of study variables in the H^2^‐registry on recommendations issued by ICHOM due to the registry's focus on patient outcomes.^35^ However, these data standards rely on expert consensus and have, to the best of our knowledge, not yet been implemented and evaluated in large clinical trials. The H^2^‐registry will serve as a first use case for application of the ICHOM standards in a multicentre HF registry.

### Outlook

The H^2^‐registry serves as a rich resource for analysing specific cardiac conditions and interventions. For instance, currently, the use of oral anticoagulation in HF patients with atrial fibrillation undergoing PCI is being investigated. Initially designed as a general CVD registry, the H^2^‐registry will be extended in the near future to include patients with atrial fibrillation and coronary artery disease as well. Moreover, the registry's infrastructure will serve as a foundation for future registry‐based clinical trials.^11^, ^12^ One trial focusing on rapid up‐titration of GDMT based on patient phenotypes is currently in preparation (PHRASE‐HF).

## Conclusions

The H^2^‐registry currently represents the largest ongoing prospective registry of HF patients in Germany. Starting in 2021, over 2300 patients have already been enrolled in 10 active study centres. With data collection based on ICHOM standards, findings from the H^2^‐registry are expected to offer an extensive and distinctive perspective on current characteristics, treatment strategies, and resultant outcomes of HF patients in Germany with specific focus on PROs.

## Conflict of interest

We declare no conflicts of interest associated with this publication.

## Funding

The H^2^‐registry is supported by AstraZeneca GmbH.

## Appendix

**Table A1:** Baseline variables

Variable ID	Variable definition
AGE	Age of the patient
SEX	Sex of the patient
DIAGCAT	Diagnostic category (aetiology of HF)
SMOKE	Smoking status
ALCOHOL	Alcohol consumption
HEIGHT	Height of the patient
WEIGHT	Weight of the patient
HOSPADM	Previous HF hospitalizations
HOSPADMNUM	Number of previous HF hospitalizations
HOSPADMTYP	Type of previous HF hospitalizations (elective vs. non‐elective)
NYHA	NYHA class
COMORBIDITIES	
AFIB	Atrial fibrillation
ISCH	Ischaemic heart disease
HYPERTEN	Arterial hypertension
VASCDIS	Vascular disease
STROKE	Stroke
DIAB	Diabetes
HYPERTHYR	Hyperthyroidism
CHRONLD	Chronic lung disease
RENDYS	Renal dysfunction
DIALYSIS	Dialysis
GASTROHAEM	Gastrointestinal bleeding
ICHAEM	Intracranial bleeding
OSA	Obstructive sleep apnoea
CANCER	Cancer
LIVER	Liver disease
EJECFRAC	Ejection fraction (category)
EJECFRACVALUE	Ejection fraction (value)
EJECDAT	Date of ejection fraction value
EJECMOD	Modality used for determining ejection fraction (echocardiography, magnetic resonance imaging, laevocardiography)
BASECREAT	Creatinine
BASEBNP	NT‐proBNP
MEDICATION	
PHARMATYPE1	Angiotensin‐converting enzyme (ACE) inhibitors
PHARMATYPE2	Angiotensin receptor II blocker (ARB)
PHARMATYPE3	Angiotensin receptor blocker/neprilysin inhibitor combination (ARNI)
PHARMATYPE4	Beta‐blocker (BB)
PHARMATYPE5	Calcium channel blocker
PHARMATYPE6	Digoxin/digitoxin
PHARMATYPE7	Other antiarrhythmic drugs
PHARMATYPE8	Diuretics
PHARMATYPE9	Hydralazine and isosorbide dinitrate
PHARMATYPE10	Ivabradine
PHARMATYPE11	Mineralocorticoid receptor antagonists (MRA)
PHARMATYPE12	Platelet inhibitors
PHARMATYPE13	Oral anticoagulation therapy (OAC)
PHARMATYPE13a	Vitamin K antagonists
PHARMATYPE13b	New oral anticoagulants (NOAC)
PHARMATYPE14	Statins
PHARMATYPE15	Sodium‐glucose co‐transporter‐2 (SGLT2) inhibitors
PHARMATYPE16	Glucagon‐like peptide‐1 (GLP‐1) receptor agonists
PHARMATYPE17	Insulin
PHARMATYPE18	Other oral antidiabetic agents
PROCEDURES	
DEVPROCEDUREACT	Device procedure during index hospitalization
DEVPROCEDUREACTTYP	Type of device procedure
DEVPROCEDUREPREV	Device procedure, previously performed
DEVPROCEDUREPREVTYP	Type of device procedure
DEVPROCEDUREDATE	Date of last device procedure
CARDSURGACT	Cardiac surgery during index hospitalization
CARDSURGACTTYP	Type of cardiac surgery
CARDSURGPREV	Cardiac surgery, previously performed
CARDSURGPREVTYP	Type of cardiac surgery
CARDSURGDATE	Date of last cardiac surgery
PCIACT	Percutaneous coronary intervention during index hospitalization
PCIPREV	Percutaneous coronary intervention, previously performed
PCIDATE	Date of last percutaneous coronary intervention
PERCVALVACT	Percutaneous valve procedure during index hospitalization
PERCVALVACTTYP	Type of percutaneous valve procedure
PERCVALVPREV	Percutaneous valve procedure, previously performed
PERCVALVPREVTYP	Type of percutaneous valve procedure
PERCVALVDATE	Date of last percutaneous valve procedure
CATHABLACT	Catheter ablation during index hospitalization
CATHABLACTTYP	Type of catheter ablation
CATHABLPREV	Catheter ablation, previously performed
CATHABLPREVTYP	Type of catheter ablation
CATHABLDATE	Date of last catheter ablation
ELECCARDACT	Direct current cardioversion during index hospitalization
ELECCARDPREV	Direct current cardioversion, previously performed
ELECCARDDATE	Date of last direct current cardioversion
LAAOCCLACT	Left atrial appendage occluder implantation during index hospitalization
LAAOCCLPREV	Left atrial appendage occluder implantation, previously performed
LAAOCCLDATE	Date of last left atrial appendage occluder implantation
ADMDATE	Admission date
ADMTYPE	Admission type
ADMAHF	Admission due to acute HF
AHFDENOVO	Admission due to de novo HF
AHFWORSE	Admission due to worsening HF
ADMCHF	Admission with chronic heart failure (all elective and non‐elective admissions except admissions for acute heart failure)
DISDATE	Discharge date
DISTYPE	Discharge type
COMPLDEVICE	Complications due to HF device therapy with specification
COMPLDEVICEDATE	Date of complication due to HF device therapy
COMPLHOSP	Nosocomial infections
COMPLHOSPDATE	Date of nosocomial infection
COMPLMED	Complication due to oral medication with specification
COMPLMEDDATE	Date of complication due to medication
COMPLMEDTYP	Substance class associated with oral medication
DEATH	In‐hospital death
DEATHDATE	Date of in‐hospital death
KCCQ‐12‐SS	KCCQ‐12 summary score
KCCQ‐12‐PL	KCCQ‐12 physical limitation
KCCQ‐12‐QOL	KCCQ‐12 quality of life
KCCQ‐12‐SF	KCCQ‐12 symptom frequency
KCCQ‐12‐SL	KCCQ‐12 social limitation
PROMIS‐MH	PROMIS GH‐10 mental health score
PROMIS‐PH	PROMIS GH‐10 physical health score
PHQ‐2	PHQ‐2 score

HF, heart failure; HF device therapy, cardiac resynchronization therapy with or without defibrillator, implantable cardioverter defibrillator therapy; KCCQ‐12, Kansas City Cardiomyopathy Questionnaire; NYHA, New York Heart Association; PHQ‐2, Patient Health Questionnaire‐2; PROM, patient‐reported outcome measure; PROMIS GH‐10, Patient Reported Outcomes Measurement Information System Global Health‐10.

**Table A2:** Follow‐up variables

Variable ID	Variable definition
SMOKE	Smoking status
ALCOHOL	Alcohol consumption
WEIGHT	Weight of the patient
HOSPADMHF	HF hospitalizations since index hospitalization
HOSPADMNUM	Number of HF hospitalizations since index hospitalization
HOSPADMTYP	Type of previous HF hospitalizations (elective vs. non‐elective)
HOSPADMCARDVASC	Hospitalization due to other cardiovascular diseases since index hospitalization with specification of the cause
NYHA	NYHA class
COMORBIDITIES	Newly diagnosed comorbidities since index hospitalization
AFIB	Atrial fibrillation
ISCH	Ischaemic heart disease
HYPERTEN	Arterial hypertension
VASCDIS	Vascular disease
STROKE	Stroke
DIAB	Diabetes
HYPERTHYR	Hyperthyroidism
CHRONLD	Chronic lung disease
RENDYS	Renal dysfunction
DIALYSIS	Dialysis
GASTROHAEM	Gastrointestinal bleeding
ICHAEM	Intracranial bleeding
OSA	Obstructive sleep apnoea
CANCER	Cancer
LIVER	Liver disease
MEDICATION	Present treatment with specified substance classes at FU
PHARMATYPE1	Angiotensin‐converting enzyme (ACE) inhibitors
PHARMATYPE2	Angiotensin receptor II blocker (ARB)
PHARMATYPE3	Angiotensin receptor blocker/neprilysin inhibitor combination (ARNI)
PHARMATYPE4	Beta‐blocker (BB)
PHARMATYPE5	Calcium channel blocker
PHARMATYPE6	Digoxin/digitoxin
PHARMATYPE7	Other antiarrhythmic drugs
PHARMATYPE8	Diuretics
PHARMATYPE9	Hydralazine and isosorbide dinitrate
PHARMATYPE10	Ivabradine
PHARMATYPE11	Mineralocorticoid receptor antagonists (MRA)
PHARMATYPE12	Platelet inhibitors
PHARMATYPE13	Oral anticoagulation therapy (OAC)
PHARMATYPE13a	Vitamin K antagonists
PHARMATYPE13b	New oral anticoagulants (NOAC)
PHARMATYPE14	Statins
PHARMATYPE15	Sodium‐glucose co‐transporter‐2 (SGLT2) inhibitors
PHARMATYPE16	Glucagon‐like peptide‐1 (GLP‐1) receptor agonists
PHARMATYPE17	Insulin
PHARMATYPE18	Other oral antidiabetic agents
PROCEDURES	Performed procedures since index hospitalization
DEVPROCEDURE	Device procedure since index hospitalization
DEVPROCEDURETYP	Type of device procedure
DEVPROCEDUREDATE	Date of last device procedure
CARDSURG	Cardiac surgery since index hospitalization
CARDSURGTYP	Type of cardiac surgery
CARDSURGDATE	Date of last cardiac surgery
PCI	Percutaneous coronary intervention since index hospitalization
PCIDATE	Date of last percutaneous coronary intervention
PERCVALV	Percutaneous valve procedure since index hospitalization
PERCVALVTYP	Type of percutaneous valve procedure
CATHABL	Catheter ablation since index hospitalization
CATHABLTYP	Type of catheter ablation
CATHABLDATE	Date of last catheter ablation
ELECCARD	Direct current cardioversion since index hospitalization
ELECCARDDATE	Date of last direct current cardioversion
LAAOCCL	Left atrial appendage occluder implantation since index hospitalization
LAAOCCLDATE	Date of last left atrial appendage occluder implantation
COMPLDEVICE	Complication due to HF device therapy with specification
COMPLDEVICEDATE	Date of complication due to device therapy
COMPLMED	Complication due to medical therapy with specification
COMPLMEDDATE	Date of complication due to medical therapy
COMPLMEDTYP	Substance class associated complication due to medical therapy
DEATH	Death during FU
DEATHDATE	Date of death
DEATHTYP	Cause of death (CV death vs. non‐CV death)
DEATHSETTING	In‐hospital death/death in the ambulatory setting
KCCQ‐12‐SS	KCCQ‐12 summary score
KCCQ‐12‐PL	KCCQ‐12 physical limitation
KCCQ‐12‐QOL	KCCQ‐12 quality of life
KCCQ‐12‐SF	KCCQ‐12 symptom frequency
KCCQ‐12‐SL	KCCQ‐12 social limitation
PROMIS‐MH	PROMIS GH‐10 mental health score
PROMIS‐PH	PROMIS GH‐10 physical health score
PHQ‐2	PHQ‐2 score

CV, cardiovascular; FU, follow‐up; HF, heart failure; HF device therapy, cardiac resynchronization therapy with or without defibrillator, implantable cardioverter defibrillator therapy; KCCQ‐12, Kansas City Cardiomyopathy Questionnaire; NYHA, New York Heart Association; PHQ‐2, Patient Health Questionnaire‐2; PROM, patient‐reported outcome measure; PROMIS GH‐10, Patient Reported Outcomes Measurement Information System Global Health‐10.

**Table A3:** Oral medication at hospital discharge (index hospitalization)

Drug	Newly prescribed, *n* (%)	Pre‐existing, continued, *n* (%)	Pre‐existing, discontinued, *n* (%)
Angiotensin‐converting enzyme inhibitors (ACEi)	136 (5.8%)	548 (23.2%)	113 (4.8%)
Angiotensin receptor II blocker (ARB)	129 (5.5%)	706 (29.9%)	97 (4.1%)
Angiotensin receptor blocker/neprilysin inhibitor combination (ARNI)	126 (5.3%)	292 (12.4%)	37 (1.6%)
Beta‐blocker (BB)	274 (11.6%)	1672 (70.8%)	102 (4.3%)
Calcium channel blocker	120 (5.1%)	477 (20.2%)	97 (4.1%)
Digoxin/digitoxin	62 (2.6%)	122 (5.2%)	48 (2%)
Anti‐arrhythmics	106 (4.5%)	154 (6.5%)	31 (1.3%)
Diuretics	295 (12.5%)	1447 (61.3%)	85 (3.6%)
Hydralazine and isosorbide dinitrate	5 (0.2%)	12 (0.5%)	4 (0.2%)
Ivabradine	11 (0.5%)	33 (1.4%)	7 (0.3%)
Mineralocorticoid receptor antagonists (MRA)	279 (11.8%)	598 (25.3%)	100 (4.2%)
Platelet inhibitors	233 (9.9%)	700 (29.7%)	84 (3.6%)
Oral anticoagulation therapy (OAC)	242 (10.3%)	1104 (46.8%)	63 (2.7%)
Vitamin K antagonists	59 (2.5%)	147 (6.2%)	34 (1.4%)
New oral anticoagulants (NOAC)	226 (9.6%)	914 (38.7%)	65 (2.8%)
Statins	235 (10%)	1352 (57.3%)	60 (2.5%)
Sodium‐glucose co‐transporter‐2 (SGLT2) inhibitors	312 (13.2%)	458 (19.4%)	32 (1.4%)
Glucagon‐like peptide‐1 (GLP‐1) receptor agonists	7 (0.3%)	33 (1.4%)	5 (0.2%)
Insulin	46 (1.9%)	357 (15.1%)	23 (1%)
Other antidiabetic agents	55 (2.3%)	419 (17.8%)	34 (1.4%)

RAASi, renin–angiotensin–aldosterone system inhibitors (angiotensin‐converting enzyme inhibitors, angiotensin receptor II blocker, or angiotensin receptor blocker/neprilysin inhibitor combination).
